# Willingness to switch to injectable cabotegravir among oral PrEP users in Durban, South Africa

**DOI:** 10.4102/hsag.v30i0.3120

**Published:** 2025-10-08

**Authors:** Tanuja N. Gengiah, Vaishnavi Naidoo, Nothile Dlamini, Maseeha Khan, Snenhlahla S. Khanyile, Sithuthukile P. Nxumalo, Minenhle L. Sithole, Azraa Sultan, Felix Made

**Affiliations:** 1 Center for the AIDS Programme of Research in South Africa, Durban, South Africa; 2 Discipline of Pharmaceutical Sciences, School of Health Sciences, University of KwaZulu-Natal, Durban, South Africa

**Keywords:** HIV prevention, pre-exposure prophylaxis, cabotegravir long acting injectable, participant

## Abstract

**Background:**

Adherence to daily oral pre-exposure prophylaxis (PrEP) is challenging. Long-acting injectable cabotegravir (CAB-LA), administered every two months, may improve adherence.

**Aim:**

To assess willingness to switch from oral PrEP to CAB-LA among adult PrEP users.

**Setting:**

PrEP users were recruited from two public-sector primary health care clinics in Durban, South Africa.

**Methods:**

A cross-sectional survey assessed oral PrEP use and related challenges. After providing information on CAB-LA, participants’ willingness to switch was evaluated. Descriptive statistics summarized data, and logistic regression identified factors associated with willingness to switch.

**Results:**

Of 126 participants, the median age was 28 years (IQR: 32–33), 88.1% were women, 96.0% identified as black African, and 83.3% were isiZulu speakers. Most were heterosexual (86.5%), 72.2% had tertiary education, and 38.1% were employed. Median oral PrEP duration was 365 days. While 92.1% reported oral PrEP fit their lifestyle, only 51.6% adhered consistently. Awareness of CAB-LA was low (8.7%), but 74.0% were willing to switch to improve adherence. Concerns about CAB-LA included injection site reactions (53.9%), systemic side effects (57.2%), and cost (82.6%). Longer-term oral PrEP users (> 1 year) had lower odds of willingness to switch (aOR: 0.36; 95% CI: 0.15–0.85; *p* = 0.019).

**Conclusion:**

Although oral PrEP was lifestyle-compatible, adherence was inconsistent. Most participants expressed willingness to switch to CAB-LA, but findings should be interpreted cautiously given study limitations. Targeted education may support longer-term oral PrEP users hesitant to switch.

**Contribution:**

This study highlights adherence challenges with oral PrEP and supports interest in longer-acting injectable alternatives.

## Introduction

In 2023, there were an estimated 39.9 million people living with human immunodeficiency virus (HIV) globally, with a reported 1.3 million new infections in the same year (The Joint United Nations Programme on HIV/AIDS [UNAIDS] 2024). South Africa has the highest HIV prevalence globally, with an estimated 7.7 million people living with HIV and approximately 150 000 new infections annually with 37% of these in people under 25 years of age (UNAIDS 2024). Current HIV prevention strategies include behavioural interventions such as sexual reproductive health education, HIV counselling and testing and condom provision, alongside biomedical approaches such as syndromic treatment of sexually transmitted infections (STIs), male medical circumcision, mother-to-child transmission prevention and the provision of daily oral pre-exposure prophylaxis (PrEP) (South African National AIDS Council 2023).

Pre-exposure prophylaxis formulations registered for use, in South Africa, include tenofovir disoproxil fumarate and emtricitabine, an oral formulation that is dosed once daily, cabotegravir long-acting (CAB-LA) injectable, a bi-monthly intramuscular injection and a monthly dapivirine vaginal ring (Macdonald et al. 2025). As of March 2025, the total number of PrEP users in South Africa was reported as 1,342,154, with 99.6% on oral PrEP, 0.3% on injectable PrEP and 0.1% on the monthly dapivirine ring (PrEPWatch 2024). However, only oral PrEP is accessible to the public at a primary healthcare level and available for widespread use while the other formulations are restricted to access from demonstration projects (PrEPWatch 2024).

Despite the availability and proven effectiveness of oral PrEP in reducing risk for HIV acquisition, several studies have reported challenges with daily use including early discontinuation, poor persistence and low adherence, limiting its overall effectiveness (Mansoor et al. 2022; Marrazzo et al. 2024). Contributing factors include concerns about stigma, judgement by others, fear of violence from partners, family and community members as well as concerns about side effects, cost and effectiveness (Celum et al. 2019; Gill et al. 2020; Mansoor et al. 2022; Pillay et al. 2020; Velloza et al. 2020). In addition to oral PrEP, the dapivirine vaginal ring was conditionally recommended by the World Health Organization (WHO) in January 2021 as an additional HIV prevention option for women at substantial risk of infection (WHO 2021). While the vaginal ring offers advantages such as discretion, privacy and the avoidance of daily pill-taking – important for women facing stigma or challenges with daily adherence – it also has limitations. Some women report discomfort with vaginal insertion and find the monthly reinsertion requirement burdensome (Griffin et al. 2019).

Given the challenges associated with oral and vaginal PrEP, there is growing emphasis on developing long acting and multipurpose technologies in the fields of HIV prevention and sexual and reproductive health (Quaife et al. 2018). Long-acting antiretrovirals (ARV) such as CAB-LA and lenacapavir are highly effective HIV prevention tools (Bekker et al. 2024; Delany-Moretlwe et al. 2022) that, unlike daily oral pills, offer extended dosing intervals and discreet use. These features may better align with user preferences and help overcome challenges related to poor adherence and persistence (Ogunbajo et al. 2022) when used for HIV prevention. The CAB-LA, an injectable PrEP, is a long acting ARV drug formulated to be administered intramuscularly every eight weeks (Bares & Scarsi 2022). Studies have shown CAB-LA to be more effective than daily oral tenofovir disoproxil fumarate/emtricitabine (TDF/FTC), demonstrating lower HIV incidence in both men and women with high reported acceptability of the injectable formulation (Delany-Moretlwe et al. 2022; Landovitz et al. 2021). Furthermore, a meta-analysis reviewing data from 2010 to 2021 showed a 79% reduction in HIV risk with CAB-LA compared to daily oral PrEP (Fonner et al. 2023), most likely because of a high participant acceptability and improved adherence with the injectable CAB-LA (Kerrigan et al. 2018, Tolley et al. 2020, Wara et al. 2023).

Although CAB-LA was approved in December 2022 by the South African Health Products Regulatory Authority (SAHPRA) for use in individuals at risk of HIV-1 infection in South Africa (SAHPRA 2022), its high cost and the absence of an affordable generic alternative remain major barriers to its implementation in South Africa (Jamieson et al. 2022; Spotlight 2024). Nevertheless, its potential advantages – such as enabling discreet use, reduced user dependency, longer dosing interval and superiority to oral PrEP (Delany-Moretlwe et al. 2022) make it a useful PrEP option for people at high risk of HIV.

Research to better understand oral PrEP users willingness to switch to injectable PrEP is limited in the South African context (Meyers et al. 2018; Roth et al. 2025). While market access was still being planned in South Africa, this study aimed to assess the willingness of current oral PrEP users to switch to injectable CAB-LA as an alternative prevention option to oral PrEP when it becomes available.

## Research methods and design

### Study design and setting

This cross-sectional quantitative study was conducted from 05 September 2022 to 07 October 2022 at two primary healthcare clinics in the Durban city centre, eThekwini district in KwaZulu-Natal province in South Africa.

### Participants and sampling

Participants enrolled in the study were individuals waiting for services at the HIV PrEP clinic, amenable to being approached and have the consent process initiated. Participants were eligible for the study if they were > 18 years, were on oral PrEP for at least 1 month and were willing and able to provide written informed consent. The authors used convenience sampling, with investigators stationed at the clinic on random days to recruit participants for private interviews conducted in-person. Convenience sampling was employed, as the study aimed to provide a descriptive, exploratory assessment rather than generalisable population estimates. This approach allowed for timely recruitment of current oral PrEP users within the available timeframe and resources, yielding a sufficient sample to generate meaningful insights into willingness to switch to CAB-LA.

### Sample size considerations

The sample size was guided by feasibility considerations to complete the interviews within the study timeframe. While formal sample size calculations for analytical precision were not the primary focus of this descriptive study, a sample of over 100 participants allowed for basic subgroup comparisons and provided a reasonable confidence in estimating proportions for exploratory public health research (Teare et al. 2014; Van Belle 2008).

### Questionnaire development and administration

For the questionnaire development, the authors drew on the Theoretical Framework of Acceptability (TFA) (Sekhon, Cartwright & Francis 2017), which offers a comprehensive lens for assessing how individuals perceive and engage with healthcare interventions. This framework is particularly well-suited to our research, as it distinguishes between anticipated acceptability (before using an intervention), the CAB-LA injection in this study and experienced acceptability (after use) of the oral PrEP that they were all taking, a key consideration when evaluating oral PrEP users’ willingness to switch to CAB-LA. The TFA outlines seven core constructs of acceptability: affective attitude (how they feel about it), burden (effort required), ethicality (alignment with values), intervention coherence (understanding how it works), opportunity costs, perceived effectiveness and self-efficacy. These constructs broadly informed the development of the questionnaire and provided a structured approach to understanding user perspectives on switching from daily oral PrEP to CAB-LA. Questions were was also informed by a combination of literature review, expert consensus from HIV prevention researchers and programmatic insights from providers familiar with end-user perspectives. While the questionnaire was not intended for use as a psychometric scale, each section was designed to be conceptually distinct and relevant to the study objectives. The instrument was reviewed internally for content validity and piloted among a small sample (*n* = 5) of pharmacy students, not enrolled in the study, to refine language clarity and flow. Feedback from this pilot was used to revise question wording and response options prior to ethics approval and implementation.

In addition to collecting demographic information, participants were also asked about the duration of their oral PrEP use and their reasons for PrEP initiation. To assess personal experiences with oral PrEP, including adherence, impact on daily life, side effects, perceived effectiveness and stigma, responses were measured using a Likert-type frequency scale ranging from ‘Never’ to ‘All the time’.

For injectable cabotegravir (CAB-LA), participants were first asked about their prior awareness of CAB-LA as a potential HIV prevention option. They were then provided with standardised information on CAB-LA, including product attributes, route and frequency of administration, estimated cost and potential side effects. This information was necessary to enable informed responses on the presumed acceptability of CAB-LA and to assess participants’ willingness to switch from oral PrEP when CAB-LA becomes available in the clinic. As CAB-LA is not yet accessible in South Africa outside of clinical trials and demonstration projects, providing this context was essential to accurately evaluate participants’ willingness to adopt this option.

These data were collected by the investigators on the 28-item questionnaire and responses were recorded in real-time. Each questionnaire was given a unique alphanumeric code to maintain participants’ confidentiality. Completed questionnaires were assessed for completeness, prior to ending the interview with participants and data were later manually captured in a REDCap® database for further analysis.

### Statistical considerations

Statistical analysis was conducted in STATA version 18.0. Continuous variables were presented as median and interquartile ranges (IQRs). Categorical data were described as frequencies and percentages. A binary logistic regression was fitted to investigate factors associated with willingness to switch to CAB-LA injectable from oral PrEP and its acceptability. Both univariate and multivariate logistic regression models are presented.

For the univariate model: relevant sociodemographic and behavioural variables (e.g., age, gender, education level, employment status, duration on oral PrEP, adherence, stigma) were first assessed to evaluate their association with the outcome: *willingness to switch to CAB-LA (yes/no)*. To avoid prematurely excluding potentially important predictors, all variables were retained in the multivariate model as they were supported by prior research even if not statistically significant in univariate analysis. The regression estimates were presented as odds ratios (ORs) and significance level (α) was set at 0.05.

### Ethical considerations

Prior to the implementation of the study, institutional consent was obtained from the University of KwaZulu-Natal Biomedical Research and Ethics Committee (UKZN BREC), approval number: BREC/00004375/2022. Gatekeepers’ permission was obtained from the two public sector PrEP clinics and the provincial KwaZulu-Natal Department of Health (KZ_202208_013). Written informed consent was obtained from all participants. All study data collected were anonymised and stored in secure databases.

## Results

A total of 126 participants were recruited in this study (Table 1) with a median age of 28 (IQR: 23–33) years, 111 (88.1%) participants were female, 91 (72.2%) having attained tertiary level education. The proportion of participants who were employed was 50.8% and 31.7% (*n* = 40) of those unemployed were students. The median duration on oral PrEP was 365 days (IQR:198–730), with 50.0% on oral PrEP for at least 1 year. The majority (122, 96.8%) of participants would recommend PrEP to others in need of HIV prevention.

**TABLE 1:** Participants’ characteristics (*N* = 126).

Variables	*n*	%	Median	IQR
**Age (years)**	-	-	28	23–33
**Age group**				
25 years and below	46	36.5	-	-
Above 25 years	80	63.4	-	-
**Sex**				
Female	111	88.1	-	-
Male	13	10.3	-	-
Preferred not to say	2	1.6	-	-
**Education level attained**				
Tertiary	91	72.2	-	-
Secondary	31	24.6	-	-
Primary	3	2.4	-	-
No formal schooling	1	0.8	-	-
**Occupation**				
Employed	64	50.8	-	-
Unemployed	62	49.2	-	-
**Sexual orientation**				
Heterosexual	109	86.5	-	-
Prefer not to say	9	7.1	-	-
Bisexual	6	4.8	-	-
Gay	2	1.6	-	-
**Duration on oral PrEP (days); median (IQR)**	-	-	365	198–730
**Would recommend oral PrEP to others**				
No	4	3.2	-	-
Yes	122	96.8	-	-

IQR, interquartile range; PrEP, pre-exposure prophylaxis.

**FIGURE 1:**
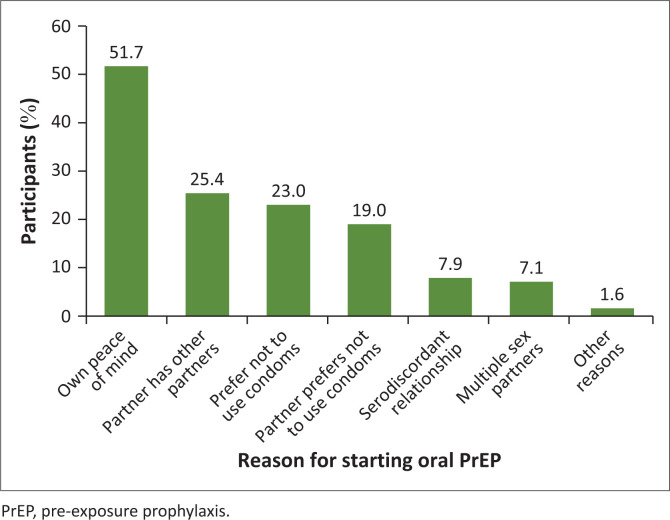
Reasons for starting oral pre-exposure prophylaxis among participants (*N* = 126).

Reasons for using oral PrEP (Figure 1) varied among participants; with 57.1% reporting starting for their own peace of mind, 25.4% knew their partners had other partners, while 23.0% and 19.0% reported that either they or their partners preferred not to use condoms, respectively. Only 7.9% of the participants who started oral PrEP were in serodiscordant relationships.

**FIGURE 2:**
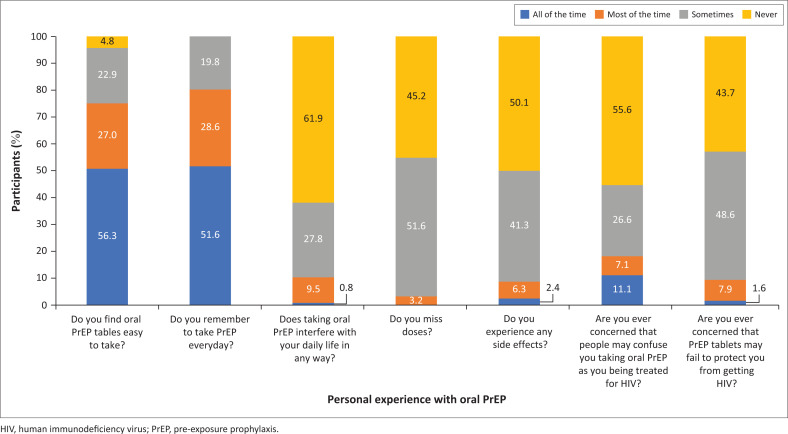
Personal experiences with oral pre-exposure prophylaxis (*N* = 126).

Participants’ personal experiences with taking oral PrEP (Figure 2) were assessed and 56.3% of the participants reported finding oral PrEP easy to take, 51.6% remembered to take their medication *all of the time* while 3.2% of the participants missed doses *most of the time*. Side effects were never experienced by 50.0% of the participants while 11.1% reported being concerned *all of the time* that people might confuse them with patients being treated for HIV.

When asked if they were ever concerned that PrEP might fail to protect against HIV infection, 1.6% of the participants reported being concerned *all of the time* and 7.9% *most of the time*.

Only 11 (9%) participants were previously aware of injectable CAB-LA PrEP. After receiving information about the CAB-LA injectable option, 88 (70%) participants indicated a preference for CAB-LA over oral PrEP.

Acceptability of CAB-LA product attributes are summarised in Table 2. Most participants found bi-monthly injections (96.8%) and clinic-based administration by a medical professional (97.6%) acceptable. However, only 79.4% found the required oral lead-in with cabotegravir tablets acceptable. The highest proportions of unacceptable responses were for the following attributes: high cost (82.5%), systemic side effects (57.1%) and injection site reactions (54.0%).

**TABLE 2:** Cabotegravir long-acting product attribute acceptability.

CAB-LA attributes	Acceptable (%)	Not acceptable (%)
Has to be injected in a clinic by a medical professional	97.6	2.4
CAB-LA is injected every 2 months	96.8	3.2
Once injected the CAB-LA is in the body for 12 months	91.3	8.7
Treatment starts with 30 days of a daily CAB tablet (lead in dose)	79.4	20.6
Injection that is administered in the gluteal muscle (buttocks) – length of the needle is 1.5 inches (3.8 cm)	76.2	23.8
CAB-LA injection causes moderate pain and tenderness at the site of injection	65.1	34.9
If HIV infection occurs while on CAB-LA then treatment must begin immediately, if not then developing resistance to HIV is possible	61.9	38.1
CAB-LA injection can also cause ISRs – nodules, induration and swelling, bruising, redness, and itching	46.0	54.0
Other common side effects can include diarrhoea, headache, and fever	42.9	57.1
CAB-LA is very expensive	17.5	82.5

HIV, human immunodeficiency virus; CAB-LA, cabotegravir long-acting; ISRs, injection site reactions.

Table 3 presents participants’ willingness to switch. When participants were asked if they would choose to switch to CAB-LA if it was *immediately available* to them that day, 57.9% of the participants were willing to switch, 23.0% were unwilling and 19.0% were unsure. The most common reason for willingness to switch was perceived ease of adherence (74.0%). Of those unwilling to switch, 48.3% cited fear of needles while 48.3% high cost of treatment. Among the participants who were unsure, 70.8% believed they did not have enough knowledge of CAB-LA to decide on the spot.

**TABLE 3:** Participants willingness to switch to cabotegravir long-acting and reasons for choice (*N* = 126).

Yes (*n* = 73) (58%)	*n*	%	No (*n* = 29) (23%)	*n*	%	Unsure (*n* = 24) (19%)	*n*	%
Adherence would be easier	54	74.0	Fear of needles	14	48.3	I don’t have enough knowledge about Cabotegravir	17	70.8
Never miss a dose	50	68.5	Cost is too high	14	48.3	Fear of adverse reactions	10	41.7
It’s safe, effective and requires less frequent administration	32	43.8	Fear of adverse reactions	12	41.4	Fear of needles	8	33.3
Fear taking tablets for PrEP, might cause the stigma that I am HIV positive	8	11.0	I don’t know enough about cabotegravir	10	34.5	Fear that injectable PrEP might fail and cause me to contract HIV	7	29.2
Fear that oral PrEP might fail to prevent HIV infection	6	8.2	Fear that the injectable might fail and cause me to contract HIV	4	13.8	Personal reasons	3	12.5
-	-	-	Personal reasons	3	10.3	Once injected the injection remains in my body for a long-time	3	12.5
-	-	-	Other	2	6.9	Other	2	8.3

HIV, human immunodeficiency virus; PrEP, pre-exposure prophylaxis.

Variables associated with willingness to switch from oral PrEP to CAB-LA are presented in Table 4. Individuals on PrEP for more than a year had significantly lower odds of willingness to switch to CAB-LA (adjusted odds ratio [aOR]: 0.36; 95% confidence interval [CI]: 0.153–0.845). Apart from duration on oral PrEP, no other variables showed a statistically significant association with willingness to switch to CAB-LA.

**TABLE 4:** Variables associated with willingness (yes vs no/unsure) to switch to cabotegravir long-acting from oral pre-exposure prophylaxis.

Variable	Univariable analysis			Multivariable analysis		
	OR	*p*	95% CI	aOR	*p*	95% CI
**Gender**						
Male	1 (ref)	-	-	1 (ref)	-	-
Female	0.82	0.742	0.252; 2.666	0.84	0.807	0.229; 3.140
**Age group**						
25 years and below	-	-	-	-	-	-
Above 25 years	1.26	0.536	0.605; 2.621	2.10	0.175	0.717; 6.188
**Education level attained**						
Secondary	1 (ref)	-	-	1 (ref)	-	-
Tertiary	0.75	0.488	0.338; 1.678	0.75	0.544	0.298; 1.889
**Occupation**						
Unemployed	1 (ref)	-	-	1 (ref)	-	-
Employed	0.86	0.697	0.427; 1.763	0.64	0.337	0.260; 1.585
**Missing doses** †						
Never	1 (ref)	-	-	1 (ref)	-	-
Sometimes	1.30	0.463	0.640; 2.656	1.14	0.762	0.465; 2.836
**Easy to take oral PrEP**	-	-	-	0.76	0.280‡	0.466; 1.246
All the time	1 (ref)	-	-	1 (ref)	-	-
Never	4.34	0.190	0.482; 39.073	6.20	0.128	0.590; 65.255
Sometimes	0.99	0.989	0.324; 3.031	1.35	0.671	0.332; 5.526
Most of the time	1.59	0.280	0.684; 3.702	1.55	0.388	0.572; 4.201
**Confuse PrEP for HIV treatment**	-	-	-	0.82	0.464‡	0.493; 1.380
Never	1 (ref)	-	-	1 (ref)	-	-
Sometimes	1.23	0.624	0.526; 2.907	1.57	0.374	0.579; 4.257
Most or all the time	0.64	0.370	0.251;1.670	0.47	0.197	0.150; 1.475
**Duration on oral PrEP**						
One year	1 (ref)	-	-	1 (ref)	-	-
More than 1 year	0.45	0.034	0.222; 0.941	0.36	0.019	0.153; 0.845
**Recommend PrEP**						
No	1 (ref)	-	-	1 (ref)	-	-
Yes	1.39	0.745	0.189; 10.211	1.43	0.739	0.171; 12.064

OR, odds ratio; CI, confidence interval; aOR, adjusted odds ratio; HIV, human immunodeficiency virus; PrEP, pre-exposure prophylaxis.

† , Missed doses most of the time, *n* = 4 combined with sometimes missed doses, *n* = 65.

‡ , Global-*p*-value for the overall effect and association.

Variables associated with CAB-LA acceptability are presented in Table 5. Higher odds of CAB-LA acceptability were observed among those with tertiary education, employed individuals and participants who sometimes missed oral PrEP doses, although these differences were not statistically significant. Lower acceptability was also observed among individuals on PrEP for over a year and those willing to recommend oral PrEP to others.

**TABLE 5:** Variables associated with cabotegravir long-acting acceptability.

Variable	Univariable analysis			Multivariable analysis		
	OR	*p*	95% CI	aOR	*p*	95% CI
**Gender**						
Male	1 (ref)	-	-	1 (ref)	-	-
Female	0.23	0.174	0.029; 1.892	0.25	0.214	0.029; 2.209
**Age group**						
25 years and below	-	-	-	-	-	-
Above 25 years	0.83	0.679	0.351; 1.977	0.52	0.301	0.1572; 1.771
**Education level attained**						
Secondary	1 (ref)	-	-	1 (ref)	-	-
Tertiary	1.15	0.756	0.468; 2.841	1.12	0.815	0.407; 3.125
**Occupation**						
Unemployed	1 (ref)	-	-	1 (ref)	-	-
Employed	1.48	0.350	0.648; 3.384	1.65	0.325	0.608; 4.481
**Missing doses** †						
Never	1 (ref)	-	-	1 (ref)	-	-
Sometimes	1.53	0.309	0.672; 3.492	1.98	0.208	0.682; 5.791
**Easy to take oral PrEP**	-	-	-	1.06	0.818‡	0.610; 1.866
All the time	1 (ref)	-	-	1 (ref)	-	-
Never	1.82	0.593	0.200; 16.660	1.72	0.672	0.160; 17.003
Sometimes	0.73	0.607	0.221; 2.414	0.55	0.267	0.084; 1.983
Most of the time	2.11	0.175	0.716; 6.270	1.93	0.277	0.575; 6.848
**Confuse PrEP for HIV treatment**	-	-	-	0.97	0.937‡	0.547; 1.743
Never	1 (ref)	-	-	1 (ref)	-	-
Sometimes	1.55	0.401	0.553; 4.382	1.24	0.718	0.386; 3.983
Most or all the time	0.98	0.972	0.335; 2.870	0.63	0.464	0.183; 2.167
**Duration on oral PrEP**						
One year or less	1 (ref)	-	-	1 (ref)	-	-
More than 1 year	0.80	0.605	0.354; 1.830	0.91	0.851	0.358; 2.330
**Recommend PrEP**						
No	1 (ref)	-	-	1 (ref)	-	-
Yes	1.06	0.955	0.107; 10.675	0.55	0.635	0.049; 6.246

OR, odds ratio; CI, confidence interval; aOR, adjusted odds ratio; HIV, human immunodeficiency virus; PrEP, pre-exposure prophylaxis.

† , Missed doses most of the time, *n* = 4 combined with sometimes missed doses, *n* = 65.

‡ , Global-*p*-value for the overall effect and association.

## Discussion

Daily oral PrEP is a safe, effective and affordable method for HIV prevention; however, challenges with consistent adherence have reduced its overall effectiveness in sub-Saharan Africa (Mansoor et al. 2022; Marrazzo et al. 2024). In this study, majority (70.0%) of oral PrEP users attending local primary healthcare clinics in Durban, South Africa, indicated a preference for CAB-LA over oral PrEP when presented with information on the intervention, with 58.0% willing to switch if CAB-LA was made available to them immediately. This is unsurprising because while participants reported a high overall acceptability for oral PrEP and found it easy to incorporate into their daily lives, only 51.6% remembered to take their doses daily, implying adherence challenges with increased risk for breakthrough infection. Comparatively, in other contexts willingness or intention to switch to CAB_LA was higher, ranging from 66.0% to 80.0% among oral PrEP users (Meyers et al. 2018; Roth et al. 2025).

Injectable PrEP, which reduces reliance on users to remember to take daily medication and allows for flexibility during periods of lower HIV risk, would be a useful addition to HIV prevention options in a high-incidence setting such as South Africa. Along with CAB-LA, given the excellent efficacy outcomes from recent trials of lenacapavir injection (Bekker et al. 2024) currently under medicines regulator review, it is likely that injectable PrEP will be available in South Africa within the next 12–18 months. This study was able to identify potential challenges with facilitating uptake of long-acting injectable PrEP when it becomes available for use in public sector clinics. An important finding was that participants who had established a stable routine with oral PrEP, having used it for over a year, were less inclined to switch to CAB-LA. Their familiarity with and possible perceived effectiveness of their current regimen may contribute to a reduced motivation to adopt a new PrEP modality (Quaife et al. 2018). This group represents an important population that should be identified early, provided with comprehensive information, given time to consider their options, and supported in choosing to remain on oral PrEP if they wish. The authors also found a general lack of knowledge and awareness about CAB-LA, with only 11.0% of participants having previously heard of it.

Participants appeared to have a high perceived effectiveness of oral PrEP and in this study the vast majority would recommend it to others requiring protection from HIV acquisition. After receiving information about CAB-LA, 70.0% of participants expressed a preference for it over oral PrEP; however, only 58.0% would actually switch if CAB-LA was immediately available while others remained unsure or hesitant to switch. Their response reflects a classic intention–behaviour gap – where individuals may express positive attitudes or preferences towards a new option (CAB-LA) but do not translate that into immediate action or behavioural commitment (actually switching) (Ajzen 2011). Participants often cited the reasonable need for additional information or concerns about overcoming their fear of needles before deciding. These barriers – and others expressed by participants – highlight the need to address knowledge gaps by distributing information, education, and communication tools widely within health facilities offering PrEP. Raising awareness, promoting community acceptance and combating myths and misinformation will be critical to supporting informed decision-making around long acting PrEP options (Castor, Meyers & Allen 2020; Evens et al. 2024; Moyo et al. 2022).

Concerns about daily oral PrEP being mistaken for antiretroviral therapy were reported by participants based on their personal experiences. This finding is consistent with other studies that have highlighted challenges with low adherence to oral PrEP, often influenced by stigma, the daily commitment required, forgetfulness, and concerns about maintaining privacy (Muhumuza et al. 2021). The stigma associated with HIV is still prevalent within our communities today and can often lead to individuals failing to seek help (Mahlalela et al. 2024). Some of the misconceptions include that individuals taking oral PrEP are promiscuous or have multiple partners and the mistaken belief that those on oral PrEP are HIV positive and taking ART (Muhumuza et al. 2021). The social and psychological effects of stigma can lead individuals to struggle with adherence to their oral PrEP regimens, miss monthly medication collections and feel uncomfortable seeking support to protect themselves from HIV, ultimately resulting in poor persistence. In contrast, CAB-LA, administered as an injection every eight weeks, and LEN, given every 6 months, offer more discreet alternatives to daily oral PrEP. These options may be preferable for individuals seeking to avoid the stigma associated with PrEP use and could potentially encourage greater uptake (Makoni et al. 2024).

Pre-exposure prophylaxis uptake in implementation programmes in South Africa is reported to be higher among women of reproductive age than men (Milimu et al. 2024) and in an analysis of 11 882 clients initiated on PrEP between January 2019 and October 2022 in Gauteng, the Eastern Cape and KwaZulu-Natal province, similar to this study, 81.9% were women (Martin et al. 2025). Prior research indicates that many women in sub-Saharan Africa favour long-acting injectable PrEP, possibly because of their familiarity with and common use of injectable contraception and the need to use PrEP without knowledge or consent of others (Laher et al. 2020; Quaife et al. 2018). While some reasons for not wanting to switch from oral PrEP included fear of needles (48.3%), fear of adverse events (41.4%), there is evidence from early CAB-LA acceptability studies, in similar populations, which indicate high acceptability and future interest in using CAB-LA after actual firsthand product use (Tolley et al. 2020).

When participants were provided with information about CAB-LA, most of its attributes – including administration by a medical professional, possible side effects, and need for treatment initiation if HIV is acquired – were found to be acceptable. However, 82.5% of participants considered the anticipated high cost of the drug unacceptable, which was unexpected given that PrEP is provided free of charge through government-funded clinics. This response reflects a strong sense of social responsibility among participants and highlights a public expectation for the judicious use of government resources. The pricing of CAB-LA in South Africa is a contentious issue (Spotlight 2024) and an ongoing access challenge for CAB-LA implementation in African countries where costs will have to be reduced substantially for it to be cost-effective (Jamieson et al. 2022). It has been suggested that sub-Saharan African countries collectively advocate for drug developers and patent waivers to enable local production (Mgodi et al. 2023).

This study has several limitations that limit the generalisability of its findings. As the study relied on non-probability sampling, the sample size is small and the findings may not be generalisable to all oral PrEP users; however, they provide valuable exploratory insights into willingness to switch to CAB-LA. The questionnaire content was based on similar surveys that assess product attributes, although it did utilise Sekhon et al.’s TFA to derive the content. A further limitation is that formal psychometric analyses (e.g., internal consistency via Cronbach’s alpha, test-retest reliability, or factor analysis) were not conducted, as the tool was not designed for longitudinal measurement or to serve as a standardised scale. In-depth interviews and/or questions were not included in the interview and could have provided more insight into participant responses. While a substantial proportion of participants reported willingness to switch to CAB-LA, these responses should be understood as indicative rather than definitive, given the cross-sectional, descriptive nature of the study and its inherent limitations.

The rollout of CAB-LA outside of clinical trials in sub-Saharan Africa began in Zambia in February 2024, which also allowed for the evaluation of implementation strategies to optimise future strategies and limit challenges. Logistical challenges such as shortages of medical equipment, point-of-care tests as well as gaps in training among healthcare professionals were experienced. However, strategies to address these challenges are being designed and implemented, and policies and guidelines are being updated, to ensure smooth scale-up (Global PrEP Learning Network 2024). As of July 2024, South Africa was scheduled to receive 231 000 doses of CAB-LA as a donation from the United States Presidents Emergency Plan for AIDS relief with the intention of rolling out injectable PrEP in state clinics and hospitals to reduce the transmission of HIV in South Africa. At the time of writing this article, this is yet to materialise. CAB-LA, if implemented at scale, could potentially reduce the transmission of HIV by 52 000 new infections each year, almost a third of the annual incidence in the region (Jamieson et al. 2022).

## Conclusion

Although oral PrEP generally aligned with participants’ lifestyles and was viewed positively, adherence among users in this study was suboptimal. The CAB-LA was perceived as a promising alternative because of its bi-monthly dosing and ease of use, with many participants indicating a willingness to switch. However, this reported willingness must be interpreted with caution. The study’s cross-sectional design, the lack of a validated data collection instrument, and structured presentation of information about CAB-LA may have introduced bias, including potential overestimation of acceptability. Furthermore, longer-term oral PrEP users expressed more hesitation, highlighting the need to explore behavioural inertia, concerns around side effects, and fear of injections. High cost and limited access also remain major barriers. Future studies using validated tools and more robust designs are needed to better understand the true acceptability and likely uptake of CAB-LA. Nonetheless, these preliminary findings suggest that targeted education and supportive messaging may play a critical role in preparing users for the introduction of long-acting injectable PrEP in South Africa.
